# A Case of Metastatic Uterine Tumor Originating from Small-Cell Lung Cancer (SCLC) Mimicking Uterine Sarcoma

**DOI:** 10.1155/2021/1809017

**Published:** 2021-07-24

**Authors:** Mariko Fujima, Yoichi Kobayashi, Momoe Watanabe, Hiromi Shibuya, Hironori Matsumoto, Yoshiko Nishigaya, Mai Momomura, Shinya Yoshiike, Kiyotaka Nagahama, Junji Shibahara, Atsushi Suzuki

**Affiliations:** ^1^Department of Obstetrics and Gynecology, Kyorin University School of Medicine, Japan; ^2^Department of Pathology, Kyorin University School of Medicine, Japan; ^3^Department of Obstetrics and Gynecology, Kosei Hospital, Japan

## Abstract

Metastatic uterine tumors originating from extragenital cancers are a rare clinical occurrence. We report a case of metastatic uterine cancer derived from small-cell lung cancer (SCLC) that necessitated surgical treatment. The patient was a 59 y/o female who had undergone chemotherapy for stage IIIB SCLC. A 15 cm uterine tumor lesion was initially detected on CT scans. The patient had previously been diagnosed with uterine fibroids, but compared to the most recent CT scans taken one and a half months earlier, imaging diagnosis revealed a sudden increase in the size of the tumor when compared to the 8 cm myoma fibroid noted previously. Additional work-up with MRI scans revealed T2-enhanced images of a tumor that had almost completely invaded the myometrium; the tumor presented with marked diffusion-weighted enhancement, and a flow void was noted within the tumor. A differential diagnosis of uterine sarcoma was considered, but due to the lack of focal hemorrhage or necrosis findings on MRI imaging, the possibility of differential diagnosis of metastatic SCLC was also noted. As the patient was experiencing abdominal symptoms including abdominal distension and tenderness due the tumor, a simple hysterectomy and bilateral salpingo-oophorectomy were performed to palliate the symptoms. During the surgical procedures, intra-abdominal findings noted peritoneal dissemination while intraoperative cell cytology diagnosis of ascites revealed small-cell cancer. The final histopathological diagnosis likewise revealed metastatic small-cell cancer from the primary lung cancer. The clinical status of the lung cancer was evaluated as progressive disease (PD), and a change in chemotherapy regimen was necessitated. Further disease progression was noted on CT scans at 2 and a half months after surgery, and with gradual systemic disease progression, the patient died of disease at 3 months postsurgery. Initial evaluation of rapidly enlarging uterine tumors should include a differential diagnosis of uterine sarcoma; additionally, it is necessary to also consider the rare possibility of metastatic disease as in the present case with a clinical history of extragenital malignancy.

## 1. Introduction

Metastatic uterine tumors originating from extragenital cancers are rare [1–3], while even more rare are uterine metastases from lung cancer [4]. We report a case of a rapidly enlarging uterine tumor discovered during treatment of lung cancer, which, while requiring a differential diagnosis from uterine sarcoma, was eventually diagnosed as metastatic uterine cancer derived from small-cell lung cancer (SCLC).

## 2. Case Presentation

The patient was 59 y/o female, gravida 3 para 3 with menopause at 55 y/o. She had been diagnosed with stage IIIB SCLC (cT2aB3N0) and had been undergoing chemotherapy and radiation treatment. Radiation treatment consisted of 60 Gy to the mediastinum while chemotherapy had been initiated with cisplatin (80 mg/m^2^) + etoposide (100 mg/m^2^); however, after 11 courses, lymph node swelling to the supraclavicular nodes was noted and subsequent chemotherapy regimen was changed to amrubicin. Additional swelling of the supraclavicular lymph nodes after 5 additional courses necessitated a further change in chemotherapy to carboplatin + paclitaxel.

One year and 10 months following initiation of chemotherapy, the patient noted increasing lower abdominal pain and an enhanced CT scan was performed. Initial imaging diagnosis demonstrated an infection of a degenerated uterine fibroid which was treated conservatively with antibiotics. An increase in LDH was noted on biochemical profile after 2 months, while a follow-up CT revealed an increase in size of the uterine tumor, leading to a gynecologic consultation. At initial pelvic examination, a uterine tumor of neonatal head size with moderate pelvic motility as well as slight serous-yellowish vaginal discharge was noted.

Hematological examinations revealed a complete blood count consisting of Hb 9.0 g/dl, Plt 12.1 × 10,000/*μ*l, WBC 6000/*μ*l, and biochemical profiles consisting of CRP 2.34 mg/dl, LDH 4100 U/l, D-dimer 9.8 *μ*g/ml, and NSE 733 ng/ml. Uterine cervical and endometrial cytology were both negative.

**Figure 1:**
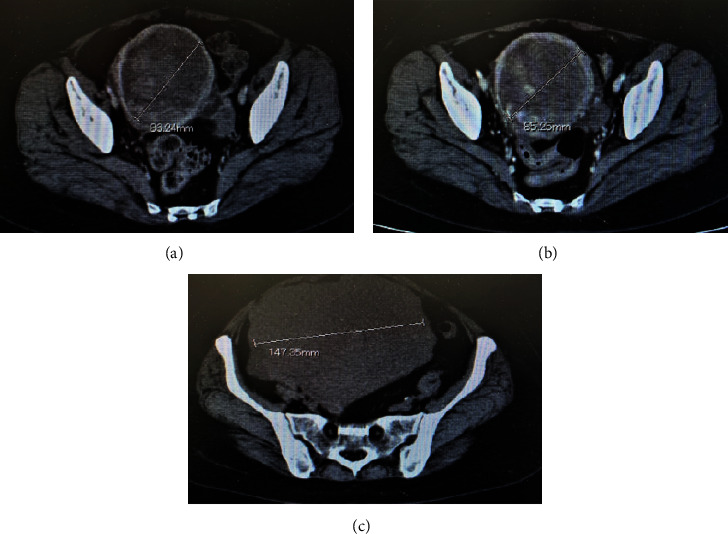
Preoperative pelvic CT findings: (a) 1 year and 6 months after initiation of treatment: myoma fibroids were noted; (b) 1 year and 10 months after initiation of treatment: the patient experienced abdominal pains, and imaging diagnosis demonstrated an infection of a degenerated myoma uteri fibroid; (c) 2 years after initiation of treatment: elevated serum D-dimer and LDH were noted, and imaging diagnosis noted a differential diagnosis of uterine sarcoma or uterine metastasis from lung cancer.

**Figure 2:**
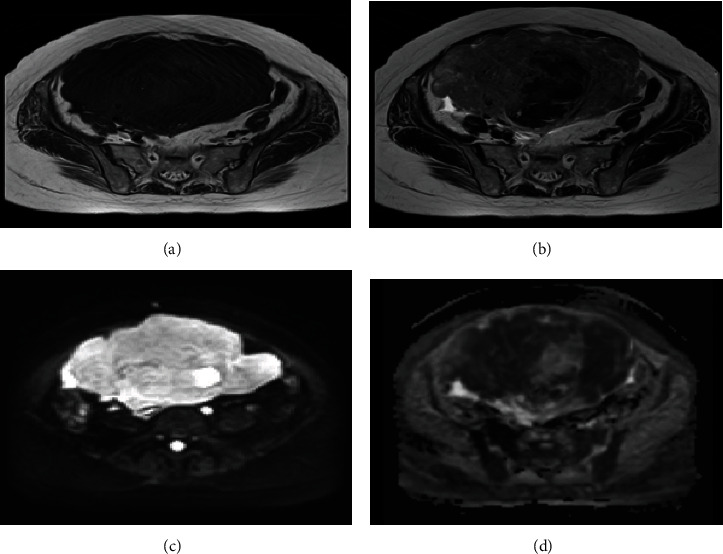
Pelvic MRI imaging: (a) T1-enhanced images; (b) T2-enhanced images; (c) ADC map b = 1000; (d) diffusion-weighted images. The tumor demonstrated moderate to strong T2-enhanced images; marked diffusion-weighted enhancement was noted on the tumor that had almost completely invaded the uterine myometrium.

**Figure 3:**
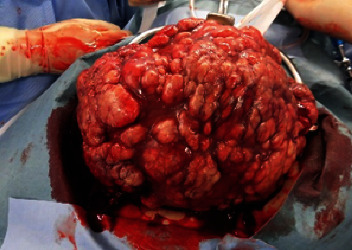
Macroscopic finding during surgery. Large diffuse nodular disseminated tumors were noted on the serosal surface of the uterus.

On CT imaging diagnosis, a sudden increase in the size of the uterine tumor compared to CT scans taken 6 weeks earlier was observed (Figure 1), but no other apparent metastatic lesions were noted. Pelvic MRI revealed multiple uterine fibroid nodules, as previously diagnosed, were noted. The tumor demonstrated T2-enhanced images and also presented with marked diffusion-weighted enhancement, and a flow void was noted within the tumor. The endometrium as well as cervical epithelium was intact, and there was also a lack of focal hemorrhage or necrotic findings (Figure 2). Based upon these findings, primary uterine sarcoma was considered, but the possibility of differential diagnosis of metastatic SCLC was also noted. Due to the increasing abdominal symptoms, including abdominal distension and tenderness due to the tumor, a simple hysterectomy and bilateral salpingo-oophorectomy were performed. Intra-abdominal findings revealed extrauterine dissemination consisting of large nodular serosal spread, and multiple sites of peritoneal dissemination were likewise noted. Macroscopically, the uterine endometrium was smooth and there was no spread of the uterine tumor to either the adnexa or to the uterine cervix (Figure 3). Ascitic cytology was class V, assumed small-cell carcinoma.

**Figure 4:**
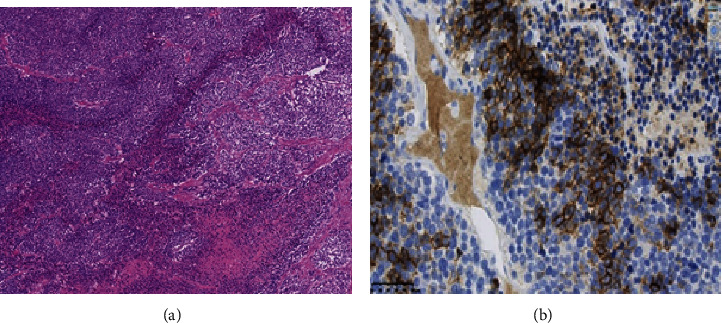
Postoperative pathological findings: (a) HE stain: tumor cells with increased chromatin, enlarged nuclei, lightly acidic cytoplasm, and increased N/C ratios were noted to proliferate in a solid nest. Localized geographic necrosis was also observed. (b) Immunohistochemical finding: the tumor demonstrated positivity for neurosecretory tumor profiles including partial positivity for CD 56, slight positivity for synaptophysin, and negativity for chromogranin A. The immunohistochemical profile was similar to that of the primary lung cancer.

Histopathological diagnosis revealed the tumor was noted to have invaded almost the entire myometrium of the uterus and demonstrated tumor proliferation with marked lymph vascular space invasion. The tumor demonstrated invasion to close to the serosal surface and localized invasion to the endometrial cavity while no cervical invasion was noted. Disseminated abdominal lesions demonstrated similar pathological features to the uterine tumor. The endometrium was mostly atrophic. Immunohistochemical profiles of the tumor revealed partial positivity to CD56, slight positivity to synaptophysin, negativity to chromogranin A, positivity to NSE, partial positivity to AE1/3 and EMA, and negativity to vimentin. Based upon these findings, the final histopathological diagnosis was metastatic small cell carcinoma of the uterus originating from the primary lung cancer (Figure 4).

The clinical status of the lung cancer was evaluated as progressive disease (PD), and although chemotherapy regimen was changed to carboplatin + irinotecan, 2 and a half months after surgery, further disease progression was observed on CT scans consisting of multiple liver metastases, increasing peritoneal dissemination lesions, and increasing pleural effusion. The patient died of disease at 3 months postsurgery.

## 3. Discussion

Metastatic uterine tumors are rare and comprise only 0.001-0.1% of all malignant uterine tumors [1]. A report of 63 cases of extragenital cancers metastasizing to genital organs found the following breakdown in origin: breast (42.9%), colon (17.5%), gastric and pancreatic (11.1%), gall bladder and lung (4.8%), malignant melanoma and bladder (3.2%), and thyroid (1.6%) [2]. Metastases from lung cancer to the female genital tract are extremely rare. Recently, Sevinyan et al. reported there were only 6 cases of lung cancers metastasizing to female genital tract including their case [5]. So, represent case is the seventh one, and so far, as we investigated, this is the first report of metastatic uterine tumor originating from SCLC.

The following hypotheses [6] have been described to account for the low incidence of metastatic spread from the lungs to the uterus: (1) the possibility that the sharp anatomic angle of the internal iliac artery leading to the uterine artery makes hinders hematogenic metastasis to the uterus; (2) the possibility that the contractive movements of the uterine myometrium prevent attachment of metastatic cells; (3) the possibility that the oxygen levels and pH within uterine tissue are not suitable for tumor attachment. However, none of these hypotheses have been clinically determined.

Although a malignant uterine tumor may be suspected from preoperative imaging diagnoses, the final diagnosis of metastatic tumor is most often diagnosed from postsurgical pathological specimens. In the present case, the uterine tumor did not present with focal necrotic or hemorrhagic findings on MRI imaging, but elevated NSE levels suggesting metastatic SCLC were noted, and the possibility of a metastatic tumor was strongly suspected preoperatively. If the metastatic tumor has invaded the endometrium and if there is a positive tumor biopsy on endometrial biopsy, it is possible to present with a differential diagnosis of metastatic tumor preoperatively; however, as in the present case, when the tumor has not invaded the endometrium [7], a preoperative histopathological diagnosis is often clinically difficult.

In general, metastasis to the uterus often occurs by lymphogenic or hematogenic routes, by direct invasion of peritoneal dissemination to the uterine serosa, and by retrograde invasion of malignant ascites through the fallopian tubes. We surmise that due to the peritoneal dissemination noted in the present case, metastatic spread to the uterus was most probably caused by tumor spread from dissemination. However, as the pathological findings noted a diffuse spread of the metastatic lesion throughout the uterine myometrium as well as direct serosal invasion, the possibility of hematogenic spread to the uterine myometrium must also be considered.

Sometimes, it is very difficult to determine whether surgical treatment should be indicated or not for the present case. In our case, uterine fibroma has rapidly grown in size, and also, the patient had abdominal distention and tenderness. So, in order to confirm the pathological diagnosis of uterine tumor as well as to relieve the patient's symptoms, surgical treatment was selected. When the patient presents with severe abdominal symptoms, as in our present case, aggressive surgical treatment may be indicated to improve the patient's QOL; however, there is little evidence whether removal of such bulky metastatic tumor could improve survival prognosis [4], so further evidences should be accumulated.

## 4. Conclusion

We report a case of metastatic uterine cancer originating from SCLC that presented with a clinical presentation that necessitated differential diagnosis from primary uterine sarcoma. In order to assess appropriate treatment strategies, a clinical presentation of rapidly enlarging uterine tumors should always include a differential diagnosis of primary uterine sarcoma, but when presented with a history of extragenital malignancies, it is important to also include a differential diagnosis of the rare possibility of metastatic disease.
